# Determination of Maintenance Energy Requirements for Fattening Castrated Korean Black Goats (*Capra hircus coreanae*)

**DOI:** 10.3390/ani11061543

**Published:** 2021-05-25

**Authors:** Sang-Ho Moon, Yeong Sik Yun, Na Yeon Kim, Sanguk Chung, Qi Man Zhang, Yujiao Tang, Sang-Hoon Lee, Jinwook Lee, Si Heung Sung, Mirae Oh

**Affiliations:** 1 Department of Food Science, College of Biomedical and Health Science, Konkuk University, Chungju 27478, Korea; moon0204@kku.ac.kr (S.-H.M.); narziss924@hanmail.net (N.Y.K.); zkzkdh53@naver.com (S.C.); zhangqiman@naver.com (Q.M.Z.); shsung@kku.ac.kr (S.H.S.); 2 Institute of Livestock Environmental Management, Daejeon 34068, Korea; lucyyellow@naver.com; 3 Asia Pacific Ruminant Institute, Icheon 17385, Korea; 4 School of Bio-sciences and Food Engineering, Changchun University of Science and Technology, Changchun 130600, China; yuanxi00@126.com; 5 Animal Genetic Resources Center, National Institute of Animal Science, RDA, Namwon 55717, Korea; sanghoon@korea.kr (S.-H.L.); koreatop5@korea.kr (J.L.); 6 Grassland and Forages Division, National Institute of Animal Science, RDA, Cheonan 31000, Korea

**Keywords:** Korean black goat, feeding standard, energy requirements

## Abstract

**Simple Summary:**

The energy required for fattening castrated Korean black goats was estimated using the correlation between metabolic energy intake per dietary body weight and average daily gain per dietary body weight. The Y-axis intercept value was the metabolic energy requirement for maintaining the lives of the fattening Korean black goats. It was calculated to be 108.76 kcal/kg BW^0.75^ (*p* < 0.05, r^2^ = 0.6036).

**Abstract:**

Twelve adult (10 months old) castrated Korean black goats, with an average initial body weight of 24.98 ± 3.7 kg, were used in this experiment to determine their maintenance energy requirements. Dry matter intakes (g/d, *p* = 0.945) were not affected by energy levels, but metabolic energy intake (kcal/d, *p* < 0.002) and average daily gain (g/d, *p* < 0.001) were significantly increased at higher energy levels. Nutrient digestibility was similar in the treatments, but crude fat digestibility increased with the addition of protective fat powder (*p* = 0.001). The energy required for fattening the castrated Korean black goats was estimated using the correlation between metabolic energy intake per dietary body weight and average daily gain per dietary body weight. The Y-axis intercept value was calculated to be 108.76 kcal/kg BW^0.75^ (*p* < 0.05, r^2^ = 0.6036), which was the metabolic energy requirement for maintaining the lives of the fattening Korean black goats. The estimated energy requirements of the black goat can improve specification techniques, such as the energy level and the amount of feed supply required for domestic black goats.

## 1. Introduction

Profitable feeding standards need to balance livestock nutrient requirements against the financial outlay of feed purchases made by farmers within the native environment of Korea. Optimized nutrient supply is indispensable for improving productivity, and its accuracy is an index in evaluating the competitiveness of the livestock industry. Feeding standards for livestock are necessary to establish optimal livestock growth stages and determine the appropriate amount of protein, energy, and mineral matter required. Productivity factors such as meat volume are closely related to environmental factors such as purchased feed [1]. The ratio of muscle to fat in livestock differs greatly depending on the nutrient content of the feed. The energy level of the feed for livestock may affect their growth [2]. Moreover, it is very important to determine the amount of energy required to maintain the livestock, since 50–70% of the energy level of the feed is used by the livestock for maintenance [3]. An inadequate supply of appropriate energy is closely related to breeding problems such as reproductive impairment [4]. Therefore, it is important to understand the maintenance energy requirements for rearing livestock.

Measuring metabolism using a respiratory calorie meter, comparative massacre, and energy balance is often used to estimate nutrient requirements [5]. Recently, the availability and scalability of large amounts of comparable data have encouraged researchers to use estimation methods based on correlation between daily weight gain and indirectly accumulated energy [6,7]. The latest revision by the National Research Council (NRC) [8] has adopted a regression equation estimation method for nutrient requirements.

Korean black goats (*Capra hircus coreanae*) have traditionally been consumed as special food for health that helps strengthen the immune system, but this consumption style has recently changed them to common meat in Korea [2]. Consequently, Korean black goat feeding farms have upgraded their facilities for large-scale operations and with a particular specialism, and goat numbers have increased. In order to secure stable productivity during goat farming, a standard that can accurately estimate and stabilize energy and nutrient requirements at each growth stage is needed. 

Unlike other ruminants, goats have a high ability to degrade the cell wall components of poor-quality feed and have physiological characteristics that often use nitrogen and water even under stressed conditions [9]. Therefore, a feeding standard for goats is necessary. However, so far Koreans have only studied nutrient requirements and feeding standards for major livestock animals such as beef cattle, dairy cattle, hens, and pigs, while other countries have goat feeding standards. The study to determine nutrient requirements of goats only began in the 1960s as they were thought to be of lower importance. A nutrient requirement profile was initiated by the NRC in 1981. However, most of the available goat feeding standards are difficult to use for the Korean black goat, as the information is unsuitable for this variety. Thus, a goat feeding standard appropriate for the Korean environment is essential [10].

In Korea, although many studies on goats have been published since 2005, only Kim et al. [11] have shown the effect of protein level difference in concentrated feed for Korean black goats on growth and feed utilization. Choi et al. [2] reported the energy requirements for the growth and meat quality of black goats. To the best of our knowledge, there are no studies that show the energy requirements for the maintenance of fattening Korean black goats. The aim of this research was to estimate the maintenance energy requirements for fattening castrated Korean black goats to help reduce feed costs for farming goats and to obtain basic data for establishing a goat feeding system using the regression equation estimation method.

## 2. Materials and Methods

### 2.1. Animals and Diets 

All animal experimentation protocols were approved by the Konkuk University Institutional Animal Care and Use Committee (KU19005). Twelve adult (10 months old) castrated Korean black goats, with an average initial body weight of 24.98 ± 3.7 kg, were used with Latin squares in experimental design (4 × 4) to determine their maintenance energy requirements. The experiment was performed at a test farm located in Icheon City, Gyeonggi-do, from October 28 to December 30, 2016. The experiment consisted of 4 treatments (energy levels: NRC + 0%, T1; NRC + 5%, T2; NRC + 10%, T3; NRC + 20%, T4), which were based on NRC’s recommendation of 108 kcal of body weight scaled at 75% (BW^0.75^) as a maintenance energy requirement for indigenous female and wether goats in their growth stages [8]. The experimental phase consisted of a 10-day feed adaptation period and a 5-day main experiment. During the entire experimental period, the black goat was raised in an individual breeding fence capable of separating urine and feces (1.5 × 0.8 m). The pen structure referred to a study by Cowan et al. [12]. Tent fabrics were used to maintain the inside temperature of the goat pens. During the experimental period, the experimental black goats were fed total mixed ration (TMR) feeds prepared for the treatments each at 09:00 and 17:00, and the average temperature of the total experimental period was 6.9 °C. The ingredients of the TMR are shown in Table 1. The TMR consisted of 14% crude protein (CP), 56% total digestible nutrients (TDNs), and 1791.9 kcal/kg energy. The energy levels of the experimental feed were controlled using protected fat powder that was a high-energy additive in which hardly any CP or crude fiber was contained, and did not affect other ingredients. The experimental feed was offered to the goats as dry matter, set at 2.0% of the goat body weight in order to induce the intake of the entire amount of experimental feed. Water was unrestricted for the goats.

### 2.2. Measuring Body Weights and Feed Intake 

The body weights and feed intake of the black goats were measured to estimate their maintenance energy requirements during the experiment. In the initial experiment, the initial body weight was used as the measurement value, and the body weight measured at the end of each period (T1–T4) was set as the final body weight. The amount of intake of feed was estimated by subtracting the residual quantity before the next feeding from the whole amount of the first feeding.

### 2.3. Sampling Procedures 

The feces of the black goats were collected and analyzed to investigate the digestibility of the samples. During the collection of manure, the total amount of manure per individual black goat was weighed and recorded. Fecal matter was collected in a plastic bag and vacuum-sealed. After each treatment, the total amount of feces samples collected per individual during the collection period was thoroughly mixed. The samples were then transferred to the laboratory, dried, and stored in an oven at 65 °C. The experimental diets (TMRs) were also dried and stored in an oven at 65 °C, and crushed to pass through a 1 mm screen (Thomas Scientific, Model 4, Swedesboro, NJ, USA) for further analysis.

### 2.4. Analytical Techniques 

To evaluate the feed value of the experimental diet, samples were collected during each experimental period. The results of the composition analysis of the experimental diets are shown in Table 2. The chemical compositions included dry matter (DM), crude protein (CP), ether extract (EE), crude fiber (CF), and crude ash of the experimental feed samples, which were determined using the method developed by the Association of Official Analytical Chemists (AOAC) [13]. The contents of neutral detergent fiber (NDF) and acid detergent fiber (ADF) were measured according to the method described by Van Soest [14]. The apparent nutrient digestibility of the feed in goats was assessed in vivo. During the 5 days of experimental period, feed intake and fecal discharge were measured, and digestibility was determined through that of each chemical component. 

### 2.5. Statistical Analysis 

All statistical analysis, except estimating the maintenance energy requirements, was performed by Tukey’s test using SAS software 9.3 (SAS Institute, Cary, NC, USA). The analysis used a randomly extracted whole block ANOVA to compare the results of 4 treatment groups. For estimating the maintenance energy requirements, a regression analysis by using correlation between average daily gain (ADG) and metabolizable energy intake (MEI) was performed using the PROC REG method of the SAS program. All data were calibrated using metabolic weight.

## 3. Results and Discussion

### 3.1. Intake and Growth of Fattening Castrated Korean Black Goats Fed with Different Metabolizable Energy Levels

The effect of energy levels on body weight, feed intake, and ADG on fattening castrated Korean black goats is shown in Table 3. The average initial body weight in each treatment group was 25 kg to minimize individual factors for goat weight. The DM intake of the goats did not significantly differ among T1 (633.0 ± 66.3 g/d), T2 (647.7 ± 62.7 g/d), T3 (635.5 ± 73.7 g/d), and T4 (636.0 ± 67.2 g/d) (*p* = 0.945). DM intake generally depends on the type of feeds and individual characteristics of animals. According to previous studies conducted by Ranjhan [15] and Lu [16], the DM intake of the goats by grazing site in India was estimated to be between 1.47% and 3.65% of body weight. In the case of forage, DMI has been shown to decrease with higher energy levels [17]. However, reduction in DM intake of forage due to increased energy levels was considered. The energy requirements of the black goats were being met by supplementary feed. In this study, it was considered that there was no significant difference in DM intake, because the experimental goats were fed a limited amount of TMR, whose energy levels were changed to add the protective fat powder against other feed components, such as forages or concentrated feeds. The amount of the diets was induced with the intake of DM set at 2.0% of the goat body weight in order for the experimental feed to be entirely consumed by the goats, and the entire feed was consumed in all the treatments in this study. 

We used the regression equation estimation method of the correlation between ADG and MEI [6,7]. Individual metabolism pens were used in this experiment to achieve accurate MEI measurements. The MEI per metabolic body weight of each treatment group was set to the maintenance energy requirement of growing indigenous goats as recommended by the NRC [8]. The T1 group was therefore set at 108.0 kcal/kg BW^0.75^. The T2 group was calculated at +5% to 113.4 kcal/kg BW^0.75^, the T3 group was calculated at +10% to 118.8 kcal/kg BW^0.75^, and the T4 group was calculated at +20% to 129.6 kcal/kg BW^0.75^. MEI during the experiment was therefore between 1233.9 ± 129.2 kcal/d and 1478.0 ± 165.0 kcal/d for the higher energy requirement levels (*p* = 0.002). The ADG was 16.3 ± 2.7 g/d for T1, 21.2 ± 8.1 g/d for T2, 29.5 ± 18.6 g/d for T3, and 65.9 ± 20.9 g/d for T4 (*p* = 0.001). These results show that the amount of ADG increased as MEI increased in the maintenance of the black goats in this study. In a similar study of Spanish goats, the metabolizable energy level of feed was 2.1–2.7 Mcal/kg, the ADG was 66.6–81.8 g/day [17], and the feed energy level was 2.79 Mcal/kg, demonstrating a high ADG [18].

### 3.2. Digestibility of Fattening Castrated Korean Black Goats Fed with Different Metabolizable Energy Levels

The apparent nutrient digestibility in castrated black goats fed at different energy levels is shown in Table 4. The average digestibility of DM was 59.7–61.2% in each treatment group. The TMR induced a balanced intake of forage and concentrated feeds that stabilized fermentation in the rumen and increased nutrient utilization [19,20]. Jung et al. [21] and Choi et al. [22] reported that the digestibility of TMR in black goats was 62–67%. The digestibility of each treatment showed a similar tendency, and no significant difference, excluding EE digestibility, was found (*p* = 0.001). Increased DMI in ruminants may cause digestibility in the rumen to drop because of an increased rate of ruminal passage [23]. However, in this study, DM intake was similar across the treatment groups despite the metabolizable energy levels being different, and therefore, feed digestibility was not affected. The EE digestibility was 66.9 ± 7.5% in T1, 74.0 ± 7.9% in T2, 75.2 ± 10.8% in T3, and 74.2 ± 10.0% in T4 (*p* = 0.001) in this study. Thus, digestibility was higher in the feed supplemented with protected fat powder. The EE digestibility in this study was similar to that in a report on oil being added to a concentrated feed mixture containing 7:3 alfalfa and hay, where digestibility was 63.48% for an oil-free feed, 82.88% for a soybean oil supplement, and 82.37% for a corn oil supplement [23]. Generally, ruminant feed has a total fat content of 2–5%. In actual specifications, when the fat content of the feed is above 5%, the digestion of crude fiber in the rumen decreases. When the fat content of the feed exceeds 7–8%, the rumen microbiome is altered, having a direct or indirect effect on the degrading bacteria and methanogenic bacteria. This may lead to metabolic disorders as the ruminal fermentation mechanism is changed [24,25,26]. In this study, we used protective fat powder, which protects the fat from dilution, degradation, and hydrogenation in the rumen. This maintains the rumen digestion and fermentation mechanism in the normal state and enhances lipase activity in the small intestine. 

### 3.3. Estimation of Energy Requirements for Maintenance of Fattening Castrated Korean Black Goats

**Figure 1:**
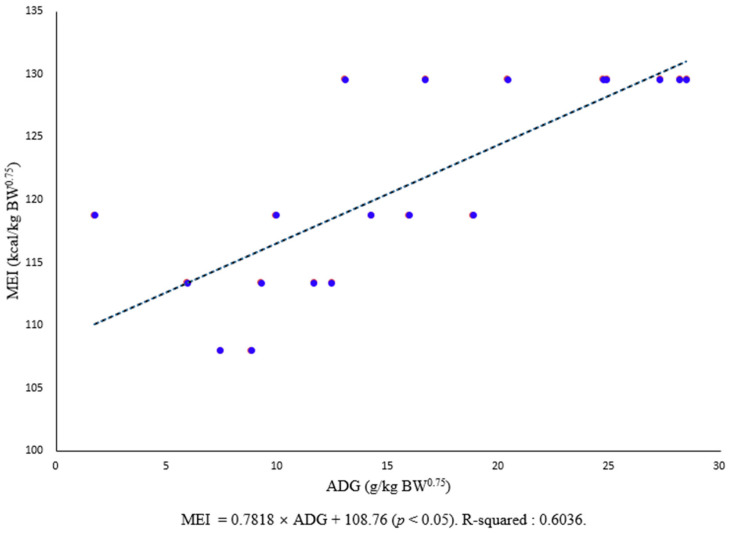
Relationship between metabolizable energy intake (MEI) (in Kcal/kg BW^0.75^) and average daily gain (ADG) (in g/kg BW^0.75^) of fattening castrated Korean black goats. Points are observed values; the line represents the regression line for all observations and describes the equation: MEI = 0.7818 × ADG + 108.76, r^2^ = 0.6036.

The results of estimating the energy requirements for fattening castrated Korean black goats using the correlation between MEI per dietary body weight (Table 3, T1, 108.0 kcal/kg BW^0.75^; T2, 113.4 kcal/kg BW^0.75^; T3, 118.8 kcal/kg BW^0.75^; T4, 129.6 kcal/kg BW^0.75^) and ADG per dietary body weight are indicated in Figure 1. The Y-axis intercept value, which is the metabolic energy requirement for the maintenance of fattening castrated Korean black goats, was calculated at 108.76 kcal/kg BW^0.75^ (*p* < 0.05, r^2^ = 0.6036). The amount of energy required for maintenance varies depending on age, sex, breed, and breeding environment [8]. It is also known that, as age increases, the maintenance requirement by body size decreases [6,7]. Metabolizable energy for maintenance requirements was summarized by the NRC, which is 117 kcal/kg BW^0.75^ for growing indigenous or local goats with weaning at 1.5 years of age [8]. In a past research by Prieto et al., the results of the experiment in which goats were fed Lucerne hay and mixed diet showed that energy requirements for maintenance are estimated to be 106.83 kcal/kg BW^0.75^ and 105.16 kcal/kg BW^0.75^ [9]. Since there was no statistical significance in the Prieto experiments, they calculated a composite regression with an energy requirement of 105.88 kcal/kg BW^0.75^ for maintenance [9]. However, the NRC recommends that energy requirements for the maintenance of indigenous goats should be 108 kcal/kg BW^0.75^ and 101 kcal/kg BW^0.75^ during growth and mature periods, respectively, for female and wether goats [8]. In Korea, where black goats are set to mature at 8 months of age, and the black goats in this experiment were 10 months old, the results of this study are similar to those of the NRC [8] for the growing stage. The difference between the energy requirement estimates from the present study and those from the study of Sahlu et al. [27] is presumably due to the number of varieties, experimental methods, and number of data points used to estimate the regression equations. In general, the estimation of nutrient requirements by regression analysis may be less accurate than that by other methods. It is difficult to know whether energy is being used for accumulation or maintenance in the body, and the effects of intestinal fullness, variety, and age may further impact interpretations. Therefore, Sahlu et al. [27] suggested that varieties and genetic characteristics need to be considered when estimating the energy requirements of goats. 

Energy requirements for maintenance also differ with seasonal and temperature changes [28,29,30]. A previous study conducted by Salah et al. [31] determined the nutrient requirements of goats to be 108.01 kcal/kg BW^0.75^ in a tropical climate and 105.90 kcal/kg BW^0.75^ in a temperate climate. Nutrient management depend on environmental temperature is very important. Livestock stress due to too high or low temperatures may cause reductions of feed intake. Therefore, feed should contain energy sources with high-energy utilization rates and should supply appropriate levels of energy. Small ruminants in tropical climates appear to have higher metabolizable energy requirements for maintenance compared with those in temperate climates.

## 4. Conclusions

The maintenance energy requirement was calculated to be 108.76 kcal/kg BW^0.75^ in this study, which is similar to the maintenance energy requirement for a tropical environment. However, the study was carried out from October 28 to December 30, during a winter climate. Goats have higher energy requirements in winter months than in other seasons to maintain their body temperature. For weight maintenance alone, higher energy supply needs to maintain body temperature in a low-temperature environment. As a result of the study, it should be considered that the maintenance energy requirements of Korean black goats can be greatly affected in the four seasons in Korea. Additionally, it can help to prevent animal feed waste in goat farms and baseline data to establish the Korean black goat feeding system. There should be further study on maintenance energy requirements of black goats according to seasonal energy needs to establish more specific information about energy requirements for the maintenance of Korean black goats.

## Figures and Tables

**Table 1:** Ingredients of the experimental total mixed ration (TMR).

Ingredients	TMR ^a^ (%)
Corn gluten feed	4.0
Lupine, grounded	2.0
Yeast culture	3.5
Wheat gluten	10.0
Corn grain, grounded	3.0
Coconut kernel meal	18.9
Soybean hull pellet	2.0
Soybean meal	3.6
Limestone	2.8
Salt	1.0
Multimix vitamin	0.3
Sodium bicarbonate	0.6
Magnesium oxide	0.3
Molasses	1.2
Water	1.5
Glycerin	0.3
Alfalfa hay	5.0
Perennial ryegrass	30.0
Klein grass hay	10
Total	100

^a^ TMR (total mixed ration) containing 14% crude protein, 56% total digestible nutrients, and 1791.9 kcal/kg energy.

**Table 2:** Ingredients of the experimental total mixed ration (TMR).

Contents ^1^	DM	CP	EE	CF	NDF	ADF	Ash
	%	----------------------------------------------------- % in DM -----------------------------------------------------					
TMR	91.9 ± 0.4	13.7 ± 0.8	6.3 ± 0.9	37.5 ± 0.4	57.4 ± 0.5	32.5 ± 0.9	8.9 ± 0.3
Protected fat	97.9 ± 0.1	0.1 ± 0.02	95.4 ± 0.4	-	-	-	12.7 ± 0.3

^1^ DM: dry matter, CP: crude protein, EE: ether extract, CF: crude fiber, NDF: neutral detergent fiber, ADF: acid detergent fiber.

**Table 3:** Effect of energy levels on body weight and feed intake in fattening castrated Korean black goats (*n* = 12).

Contents	Treatments ^1^				SEM ^2^	*p* Value ^3^
	T1	T2	T3	T4		
Initial body weight (kg)	25.8	26.5	25.8	25.9	0.55	0.959
Final body weight (kg)	26.6	26.3	26.2	26.5	0.55	0.992
Dry matter intake (g/d)	633.0	647.7	635.5	636.0	2.37	0.945
Metabolizable energy intake (Kcal/kg(BW^0.75^))	108.0	113.4	118.8	129.6	-	-
Metabolizable energy intake (Kcal/d)	1233.9 ^c^	1325.7 ^b^	1358.5 ^b^	1478.0 ^a^	3.47	<0.002
Average daily gain (g/d)	16.3 ^c^	21.2 ^b^	29.5 ^b^	65.9 ^a^	0.96	<0.001
Average daily gain (g(BW^0.75^)/d)	8.1 ^c^	9.8 ^b^	12.1 ^b^	22.9 ^a^	0.55	<0.001

^1^ T1: NRC + 0%, T2: NRC + 5%, T3: NRC + 10%, T4: NRC + 20%. ^2^ SEM: standard error of means. ^3^ The effect of feed intake level. ^abc^ Means with different superscripts within the same row are significantly different (*p* < 0.05).

**Table 4:** Apparent nutrient digestibility in castrated black goats fed diets of different metabolizable energy requirement levels.

Contents ^1^	Treatments ^2^				SEM ^3^	*p* Value ^4^
	T1	T2	T3	T4		
DM (%)	59.7	60.9	61.2	59.8	1.12	0.981
CP (% in DM)	66.2	69.3	68.6	67.9	1.09	0.881
EE (% in DM)	66.9 ^b^	74.0 ^a^	75.2 ^a^	74.2 ^a^	1.22	0.001
CF (% in DM)	72.2	72.4	73.0	73.2	0.95	0.986
NDF (% in DM)	60.3	61.1	62.4	60.9	1.10	0.966
ADF (% in DM)	58.7	59.5	62.0	59.4	1.10	0.873
Ash (% in DM)	29.2	28.6	25.3	29.0	1.38	0.937

^1^ DM: dry matter, CP: crude protein, EE: ether extract, CF: crude fiber, NDF: neutral detergent fiber, ADF: acid detergent fiber. ^2^ T1: NRC + 0%, T2: NRC + 5%, T3: NRC + 10%, T4: NRC + 20%. ^3^ Standard error of means. ^4^ The effect of feed intake level. ^ab^ Means with different superscripts within the same row are significantly different (*p* < 0.05).

## Data Availability

The data presented in this study are available on reasonable request from the corresponding author.
